# Effects of Different Postharvest Treatments on Fruit Quality, Sucrose Metabolism, and Antioxidant Capacity of ‘Newhall’ Navel Oranges During Storage

**DOI:** 10.3390/plants14050802

**Published:** 2025-03-05

**Authors:** Bo Xiong, Linlv Han, Yinghong Ou, Wenjia Wu, Jialu Wang, Junfei Yao, Yisong Li, Siyu Chen, Taimei Deng, Hongzhen Chen, Chenming Wang, Qingqing Ma, Yujing Fan, Yixuan Li, Zhihui Wang

**Affiliations:** College of Horticulture, Sichuan Agricultural University, Chengdu 611130, China; 18989192178@163.com (L.H.); 18284187563@163.com (Y.O.); 202302925@stu.sicau.edu.cn (W.W.); wangjial0321@163.com (J.W.); 19845921559@163.com (J.Y.); liyisong0629@163.com (Y.L.); csy18190735953@163.com (S.C.); dtm123692023@163.com (T.D.); 15775554829@163.com (H.C.); wangchenming0104@163.com (C.W.); 122005185923@163.com (Q.M.); fan0712150024@163.com (Y.F.); liyixuan112025@163.com (Y.L.); wangzhihui318@sicau.edu.cn (Z.W.)

**Keywords:** ‘Newhall’ navel orange, postharvest storage, antioxidant properties, sugar metabolism, citrus preservation

## Abstract

During the post-harvest storage of citrus, the flavor of fruit gradually fade. In this study, we investigated the effects of different treatments—control check (CK), heat treatment (HT), salicylic acid treatment (SA), and 1-methylcyclopropene treatment (1-MCP)—on the quality of ‘Newhall’ navel oranges, particularly focusing on sucrose metabolism and related gene expression during storage. Combining the experimental data, we compared the three different treatments with CK. The results showed that the oranges subjected to HT had a significantly higher flavonoid content (26.40 μg) and total phenolic content (19.42 μg) than those used for the CK at the late storage stage, and was also the most effective in slowing the decline in sugar, titratable acid and other indexes, followed by SA, with 1-MCP performing poorly. Quantitative results showed that the three treatments contributed to the increase in sucrose content by elevating the expression of the *SPS1* and *SPS2* genes involved in sucrose synthesis compared to the CK. However, no clear pattern was observed between the genes involved in sucrose catabolism (*SUS1* and *SUS3*) and sucrose content. These results provided a rationale for the selection of post-harvest treatments to extend the storage life and maintain the quality of ‘Newhall’ navel oranges, with broader implications for the citrus industry.

## 1. Introduction

The ‘Newhall’ navel orange, a citrus plant belonging to the *Rutaceae* family, originates from the United States and is currently cultivated in regions such as Sichuan, Chongqing, Jiangxi, and other places in China. Despite its notable resistance to storage and transportation, the fruit’s quality still deteriorates to varying degrees during post-harvest handling and distribution, thereby impacting its taste [1]. Sugar content, a critical determinant of fruit flavor, frequently becomes the focal point in research concerning postharvest storage.

Soluble sugars in citrus fruits mainly consist of sucrose, fructose and glucose, with their contents and proportions varying according to the citrus variety [2]. Sucrose is one of the most abundant soluble sugars in citrus, and serves as the main form of sugar transport in different organs [3], playing a crucial role in citrus sucrose metabolism. Sucrose phosphate synthase (SPS) and sucrose synthase (SS) are key enzymes regulating sucrose fluxes in sink tissues for sucrose accumulation in citrus [4]. A genome-wide characterization of citrus sucrose synthase revealed that SS catabolic activity was significantly correlated with sucrose content, suggesting that SS influences sucrose levels and thus sugar accumulation in the fruit [5]. SPS catalyzes the conversion of fructose-6-phosphate and UDP-Glc to sucrose-6-phosphate, which is subsequently hydrolyzed to sucrose by sucrose–phosphatase. Consequently, SPS synchronizes sucrose metabolism and accumulation [6].

In an ever-changing global marketplace with increasing competition, the citrus industry is continuously seeking new varieties of fruit to extend the marketing period, diversify and differentiate the product and, importantly, provide the fruit with a higher nutritional value and additional health-related properties [7]. The application of GRAS salts, phytopharmaceuticals and yeasts represents a promising integrated approach for managing postharvest diseases in citrus, addressing consumer concerns about the toxicity of residuals in edible fruits [8]. Mechanistic, observational and intervention studies have demonstrated that the bioactive compounds present in fruits, vegetables and whole grains prevent oxidative damage to cells by detoxifying free radicals [9], thereby minimizing the incidence of many diseases associated with metabolic syndrome, including neurodegenerative diseases, cardiovascular disease (CVD), type 2 diabetes and cancer [10]. The antioxidant properties of foods have received increasing attention as an important parameter for evaluating fruit quality. Flavonoids and total phenols play a direct role in scavenging reactive oxygen species, inhibiting lipid oxidation in vitro, enhancing the activity of antioxidant enzymes in the body and reducing peroxide formation [11]. It is important to note that the antioxidant activity of plant extracts can be determined using various methods, such as ABTS [2,2′-Azinobis-(3-ethyl-benzthiazoline-6-sulphonate)], DPPH (2,2-diphenyl-1-picrylhydrazyl) and FRAP (Ferric Reducing Antioxidant Power) [12].

Common postharvest fruit storage methods can be categorized into chemical, physical and biological preservation. There are currently three chemical fungicides commonly used for postharvest citrus preservation: benzimidazole, imidazole and bisguanidine salts [13]. Peach fruits are susceptible to cold damage during low-temperature storage. However, treatments with hot air or hot water have been shown to preserve fruit quality, reduce reactive oxygen species (ROS) and enhance antioxidant activity [14]. *Bacillus pelliculatus* HY19 can inhibit Penicillium infestations in citrus peel by releasing volatile organic compounds, thereby reducing the incidence of Penicillium and Green Mold, and decreasing the decay rate of citrus fruits [15]. For environmental and health reasons, scientists are now focusing on the development of new, environmentally friendly postharvest treatments. Yellow peel leaves show promise as a natural preservative for postharvest citrus treatments [16].

Previous studies have demonstrated that treatment with physical treatments (e.g., low temperature storage, heat treatment, etc.) or non-toxic chemical reagents (e.g., salicylic acid, 1-Methylcyclopropene (1-MCP)) can effectively preserve the freshness of citrus fruits. The decay rate and water loss rate of navel oranges stored at 5 °C were significantly lower than those stored at 26 °C [17]. Citrus fruits treated with salicylic acid (SA) maintained a better fruit firmness, total soluble solids and vitamin C (Vc) content during storage [18]. The pre-harvest application of SA alone or in combination with ascorbic acid (ASA) was effective in preventing pomegranate frost damage and maintaining pomegranate quality. It was determined that a combination of SA 250 ppm and ASA 250 ppm was the most effective for protecting plants from environmental stresses such as heavy metals, high temperatures and salinity [19]. Date palm fruits exhibited higher organoleptic attributes after 3 min and 5 min of treatment, which improved ripening and preserved nutritional quality postharvest [20]. After storage, 1-MCP-treated citrus fruits showed significantly higher levels of total phenol, antioxidant activity, flavonoids, proteins and free amino acids compared to untreated citrus fruits [21].

Research on citrus antioxidants has garnered significant attention. However, limited studies have focused on ‘Newhall’ navel orange varieties. Additionally, there is a lack of in-depth analyses on the effects of different treatments on sucrose metabolism during storage, particularly from the perspectives of enzyme activities related to sucrose metabolism and gene expression. In this study, we investigated the impact of various postharvest treatments on citrus fruit quality by evaluating antioxidant properties such as flavonoid content, physiological indexes (fructose, glucose, sucrose), and molecular parameters including the activities of enzymes associated with sucrose synthesis and gene expression. The aim was to identify a treatment that can effectively mitigate the decline in citrus fruit quality postharvest.

## 2. Materials and Methods

### 2.1. Plant Materials and Reagent Material

Newhall navel oranges were harvested from a commercial orchard in Leibo County, Sichuan Province, China, ensuring uniformity in size and color and the absence of visible damage. Fruits were transported to the laboratory for analysis at 0, 6, 18 and 30 days postharvest. Upon arrival, pulp samples were immediately snap frozen in liquid nitrogen, homogenized, and stored at −80 °C for subsequent analysis. Each analysis included three replicates, with each replicate consisting of six fruits. Salicylic acid was analytically pure, while methanol, isopropanol and acetonitrile were chromatographically pure. The RNAprep Pure Plant Total RNA Extraction Kit for polysaccharides and polyphenols was purchased from TIANGEN Biochemical Technology Co., Ltd. (Beijing, China), and the M5 Sprint qPCR RT Kit was obtained from JUHEMEI Biotechnology Co., Ltd. (Beijing, China).

### 2.2. Experimental Treatment

Control check (CK): no treatment. Heat treatment (HT): navel oranges were soaked in hot water at 50 °C for 5 min and then dried [22]. Salicylic acid treatment (SA): a 0.3 g/L salicylic acid solution was prepared, and the fruits were soaked in this solution for 20 min before drying [23]. 1-MCP treatment (1-MCP): a commercial 1-MCP solution at a concentration of 0.2 g/L was prepared, and the fruits were immersed in the solution for 3 min, followed by drying. After the treatments, the first sampling was conducted immediately and designated as Day 0. For each sampling, six to nine fruits were selected. The pulp was separated from the skin, chopped, and stored in an ultra-low-temperature freezer at −80 °C. The remaining fruits were uniformly placed in a refrigerator set at 5 ± 1 °C with a relative humidity of 90–95%. Samples were taken and processed on the 0, 6th, 18th, and 30th storage days.

### 2.3. Measurement of Flavonoids and Total Phenols

The flavonoid content was determined using the aluminum chloride colorimetric method. Specifically, 75 μL of 95% ethanol, 10 μL of 10% aluminum chloride, 10 μL of 1.0 M potassium acetate and 140 μL of distilled water were added to the extract. After 15 min of incubation, the absorbance was measured at 415 nm. A standard curve was prepared using rutin, and the results were expressed as μg/g FW (fresh weight) [24].

**Figure 1:**
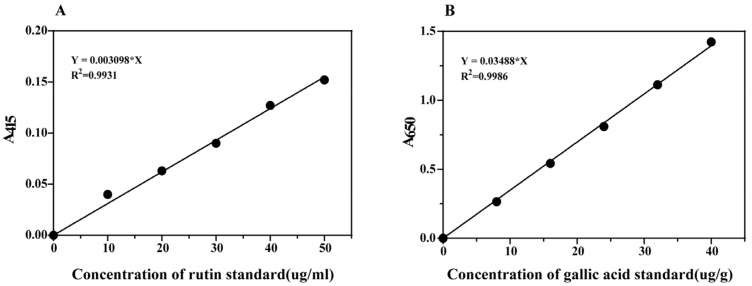
Standard curve range and R^2^ for rutin (**A**); standard curve range and R^2^ for gallic acid (**B**).

The total phenol content was quantified by the Folin–Ciocalteu phenol method. Specifically, 12.5 μL aliquots of water-soluble extracts were mixed with 250 μL of 2% sodium carbonate solution in a 96-well microtiter plate and incubated for 5 min at room temperature. Subsequently, 12.5 μL of 50% Folin–Ciocalteu phenol reagent was added, and the mixture was allowed to stand at room temperature for 30 min. The absorbance of the reaction mixture was then measured at 650 nm using a plate reader. Calibration was performed using a gallic acid aqueous solution (100–1000 μg/mL) (Figure 1) [25].

### 2.4. Measurement of DPPH, ABTS and FRAP Radical Scavenging

The experiments were primarily conducted using total antioxidant capacity assay kits (DPPH, ABTS, FRAP) provided by P Yeast Bioengineering Co., Ltd. (Wuhan, China).

### 2.5. Measurement of Fruit Weight Loss

Separately, 6 fruits were randomly selected from each treatment group and weighed using an electronic balance. Formula (1) was then used:

(1) $$Weightlessness\;(\%)=\frac{(Fruit\;quality\;before\;storage\;-\;Fruit\;quality\;before\;storage)\;}{Fruit\;quality\;before\;storage}\times 100\%$$

### 2.6. Measurement of Total Soluble Solid, Titratable Acidity, and Total Sugar

‘Newhall’ navel orange fruits were selected at each development stage to determine total soluble solids (TSSs) and titratable acidity (TA). At each developmental stage, more than 18 fruits were used for quality assessment, with three replicates established. Total soluble solids content (%) and titratable acidity (%) were measured using a digital acidometer (model Pocket PAL-BXIACID1, Atago, Tokyo, Japan) according to the manufacturer’s instructions.

Total sugars were quantified using the sulfuric acid–anthrone method [26] using Formula (2):

(2) $$Total\;sugar\;content=\frac{(Sucrose\;content\;in\;sdandard\;curve\times volume\times dilution)\;}{Volume\;of\;sample\;extract\;added\times Sample\;quality\times 10^{6}}\times 100\%$$

### 2.7. Determination of Glucose, Fructose, and Sucrose Contents

The contents of glucose, fructose and sucrose were determined according to the method described by Liu et al. [27]. Two grams of pulp were homogenized with 10 mL of double-distilled water (ddH_2_O) and incubated at 80 °C for 15 min. The mixture was then centrifuged and filtered. The resulting supernatant was analyzed using an Agilent 1260 high performance liquid chromatography system (Agilent Technologies, Santa Clara, CA, USA) equipped with a refractive index detector and an Innova NH_2_ column (4.6 mm × 250 mm, 5 μm) from Aeger Technology (Shanghai, China). The mobile phase consisted of acetonitrile–water (80:20, *v*/*v*) at a flow rate of 1 mL/min.

### 2.8. Determination of Enzyme Activities Related to Sucrose Metabolism

The activities of sucrose metabolism-related enzymes were determined with reference to the method of Zhang et al. [28]. All operations were conducted at 0~4 °C, and the absorbance values were measured immediately after adding the reaction substrate.

### 2.9. qRT-PCR Analysis

RNA was extracted from the pulp at different times of the year using the RNAprep Pure Total RNA Extraction Kit for Polysaccharide–Polyphenol Plants (TIANGEN). The extracted RNA was reverse transcribed into cDNA using the M5 Sprint qPCR RT kit (JUHEMEI) according to the instructions.

The RT-qPCR gene-specific primers are listed in Table 1, with Actin serving as the endogenous control gene. The cDNA was used as a template for amplification using the 2× M5 HiPer Dual SYBR Green Real-Time PCR Super mix kit (JUHEMEI). The amplification reaction system is detailed in Table 2, and the total volume was 12.5 µL. RT-qPCR was performed on a CFX96 Real-Time PCR Detection System (Bio-Rad, Shanghai, China).

Program: pre-denaturation at 95 °C for 2 min, denaturation at 95 °C for 5 s, annealing at 57 °C for 40 s, cycling 39 times. The melt curve method was conducted at 95 °C for 5 s, 65 °C for 1 min, followed by continuous collection at 97 °C, and cooling at 40 °C for 10 s. Each sample was analyzed in triplicate, and the relative gene expression levels were calculated using the 2^−ΔΔCt^ method.

### 2.10. Data Analysis

GraphPad Prism (version 9) was used for all data analyses, including construction and analysis of variance (ANOVA). Means and standard deviations were calculated, and the final results were presented as the mean ± SD. Post hoc multiple comparisons were conducted using the LSD test. The significance level was indicated by the *p*-value; *p* < 0.05 denoted a significant difference, and *p* < 0.01 denoted a highly significant difference between the compared groups. Data visualization was performed using Origin (version 2017), with significance levels marked in the figures.

## 3. Results

### 3.1. Changes in Flavonoids and Total Phenolic Content of Fruit Pulp During Storage

**Figure 2:**
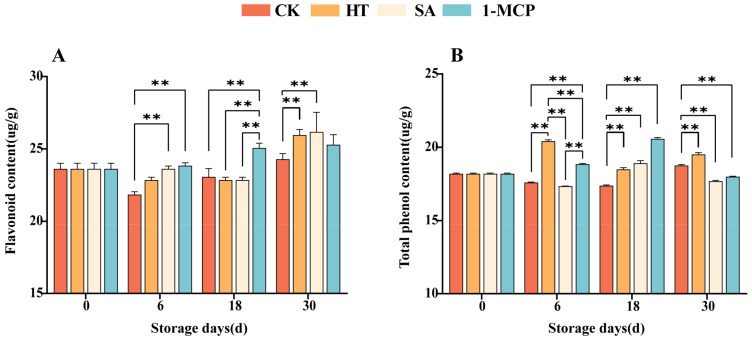
Changes in flavonoid (**A**) and total phenolic contents (**B**) of fruit pulp during storage. “**” means the result of significance analysis is highly significant (*p* < 0.01).

It was observed that the flavonoid content peaked on the 30th storage day and generally exhibited a decreasing and then increasing trend (Figure 2A). At day 30 postharvest, the flavonoid contents in the heat treatment (HT) and salicylic acid (SA) groups were significantly higher than that in the control check(CK) group (*p* < 0.01), indicating that these treatments effectively inhibited the decline in flavonoid content during the later stages of storage. Additionally, the flavonoid content in the 1-methylcyclopropene (1-MCP) group was significantly higher than that in the other three groups on the 18th storage day (*p* < 0.01), likely due to the superior effect of 1-MCP in inhibiting the decrease in flavonoid content during the middle storage stage.

Regarding total phenol content, all three treatments (HT, SA and 1-MCP) were significantly different from the CK group in the later stage (*p* < 0.01) (Figure 2B). This suggested that all three treatments effectively inhibited the decline in total phenol content, with the heat treatment performing best on 6 storage days and 1-MCP on the 18th storage day.

### 3.2. Changes in DPPH Free Radicals Scavenging, ABTS Free Radicals Scavenging and FRAP Reduction Capacity in Fruit Pulp During Storage

**Figure 3:**
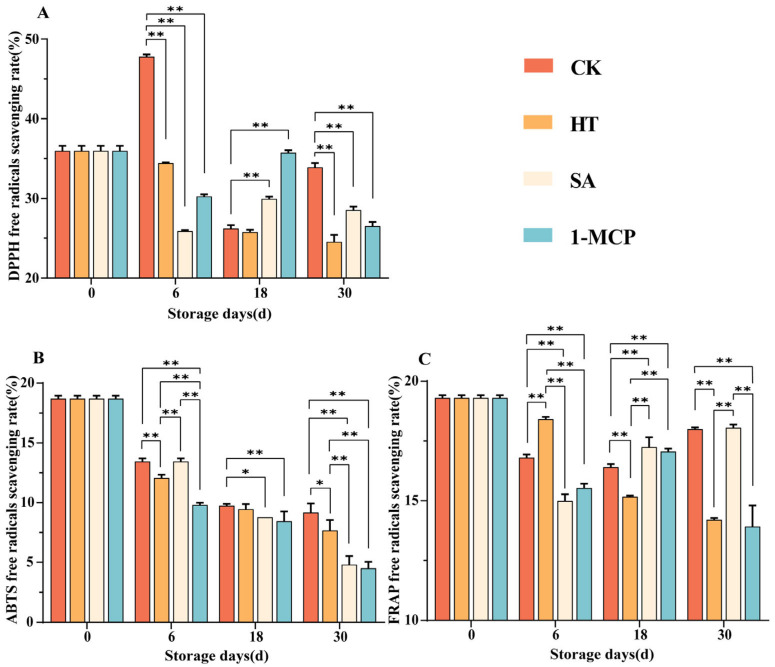
Changes in DPPH free radicals scavenging (**A**), ABTS free radicals scavenging (**B**) and FRAP free radicals scavenging (**C**) in fruit pulp during storage. “**” means the result of significance analysis is highly significant (*p* < 0.01), “*” means the result of significance analysis is significant (0.01 < *p* < 0.05).

DPPH radical scavenging, ABTS radical scavenging and FRAP total antioxidant capacity exhibited an increasing trend in the early postharvest period, followed by a gradual decrease (Figure 3). This suggested that the antioxidant performance of these samples peaked in the early postharvest period. The initial increase in antioxidant capacity was likely due to the gradual release or activation of antioxidant components, while the subsequent decline might be attributed to the depletion of these components over time. The results showed that on day 30, the ABTS radical scavenging rate of CK was 9.16%, which was significantly (0.01 < *p* < 0.05) different from HT (7.67%) and highly significant (*p* < 0.01) compared to SA and 1-MCP, suggesting that HT delayed the decline in ABTS radical scavenging rate. On day 30, the FRAP radical scavenging rate of SA was 18.05%, which was much higher than that of HT and 1-MCP, and could effectively inhibit the decrease in FRAP radical scavenging capacity.

### 3.3. Effect of Different Treatments on the Rate of Fruit Weight Loss During Storage

**Figure 4:**
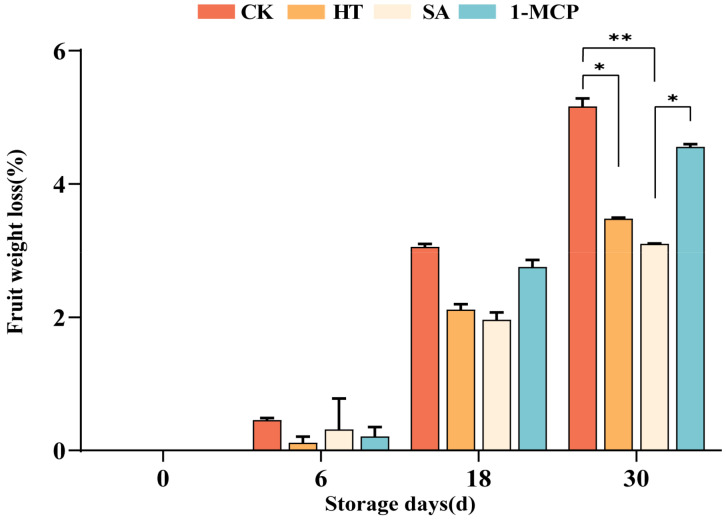
Effect of different treatments on the rate of fruit weight loss during storage. “**” means the result of significance analysis is highly significant (*p* < 0.01), “*” means the result of significance analysis is significant (0.01 < *p* < 0.05).

The rate of weight loss increased with the extension of storage time (Figure 4). On the 30th day, the weight loss rates were 3.48% for the heat treatment (HT) group, 3.10% for the salicylic acid (SA) group and 4.56% for the 1-methylcyclopropene (1-MCP) group, all of which were lower than that for the control (CK) group (5.16%). The rate in the HT group was significant (0.01 < *p* < 0.05) lower than that of the CK group, while that of the SA group was significantly (*p* < 0.01) lower than that of the CK group. There was a non-significant difference between the 1-MCP and CK groups (*p* > 0.05). These findings indicate that the heat treatment (HT) and salicylic acid (SA) treatment were more effective in retarding fruit quality decline during storage, whereas 1-MCP performed less effectively in reducing weight loss.

### 3.4. Changes in Titratable Acid and Total Soluble Solids Content of Fruit Pulp During Storage

**Figure 5:**
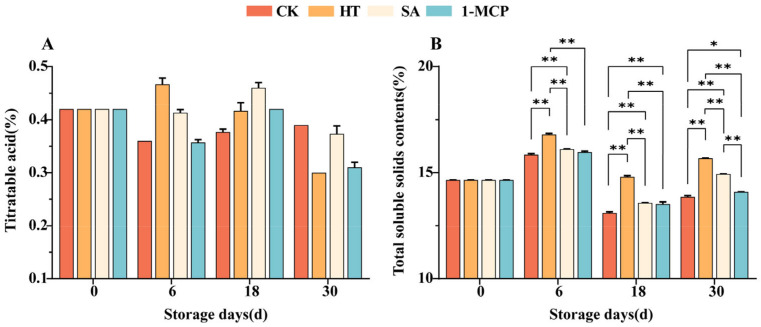
Changes in the titratable acid (**A**) and total soluble solids contents (**B**) of fruit pulp during storage. “**” means the result of significance analysis is highly significant (*p* < 0.01), “*” means the result of significance analysis is significant (0.01 < *p* < 0.05).

In the significance analysis, no significant differences were observed in the titratable acid contents among the different treatments (Figure 5A). However, our overall observations indicated that the SA treatment better stabilized the levels of titratable acids, with the CK group showing 0.42 g/100 mL of titratable acid at storage day 0, while the SA treatment maintained a level of 0.37 g/100 mL even on the 30th storage day, demonstrating minimal variation between the two groups (Figure 5B). An analysis of total soluble solids content revealed significant differences among all treatment groups. Specifically, the HT group exhibited a significantly higher total soluble solids content compared to the other three groups across all four storage periods (*p* < 0.01), indicating that the HT positively influenced fruit quality by promoting an increase in the total soluble solids content.

### 3.5. Changes in Total Sugar and Sugar Components During Storage

**Figure 6:**
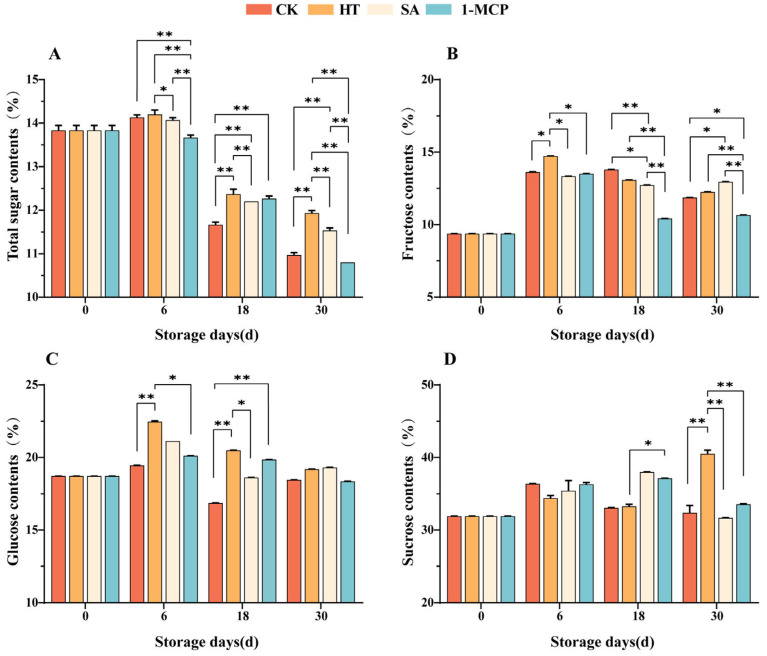
Changes in total sugar (**A**), fructose (**B**), glucose (**C**) and sucrose (**D**) contents of fruit pulp during storage. “**” means the result of significance analysis is highly significant (*p* < 0.01), “*” means the result of significance analysis is significant (0.01 < *p* < 0.05).

The total sugar content reached its peak on the sixth storage day and then gradually decreased (Figure 6A). The total sugar content in the heat treatment (HT) group was significantly higher than that in the control (CK) group at all time points except for the sixth storage day, when the difference was not significant (*p* > 0.05). During the later stages of storage, the salicylic acid (SA) treatment significantly inhibited the decrease in total sugar content, indicating that both heat treatment and salicylic acid treatment primarily delayed the decline in total sugar content during the later stages of storage. On the 30th storage day, the total sugar content in the 1-methylcyclopropene (1-MCP) treatment group was significantly lower than that in the CK group (*p* < 0.01). Overall, heat treatment and salicylic acid treatment to some extent delayed the decrease in total sugar content, with heat treatment being the most effective.

The fructose and glucose contents generally showed a trend of first increasing and then decreasing (Figure 6B). On the sixth storage day, the fructose content in the HT group was significantly higher than that in the CK group (0.01 < *p* < 0.05). However, by the 30th day of storage, only the SA group showed a significantly higher fructose content compared to the CK group (0.01 < *p* < 0.05), while the differences between the other treatment groups and the CK group were not significant (*p* > 0.05), with the results for some treatments being even lower than for the CK group, indicating that these treatments were less effective in promoting fructose accumulation. For glucose, the results for the HT group were extremely significantly higher than those for the CK group on the 6th and 18th storage days (*p* < 0.01), but the difference was not significant on the 30th day (*p* > 0.05) (Figure 6C). These results indicate that these three treatments mainly promoted glucose accumulation during the middle stage of storage but were less effective in the later stages.

The trend of the sucrose content in the CK group was similar to that of the total sugar content. On the 18th storage day, the sucrose content in the CK group was significantly lower than that in the SA group (0.01 < *p* < 0.05), and on the 30th day, it was significantly lower than that in the HT group (*p* < 0.01). In contrast, the sucrose content in the 1-MCP group did not differ significantly from that in the CK group (*p* > 0.05), indicating that heat treatment and salicylic acid treatment promoted sucrose accumulation during storage (Figure 6D).

### 3.6. Changes in the Activities of Enzymes Related to Sucrose Metabolism

**Figure 7:**
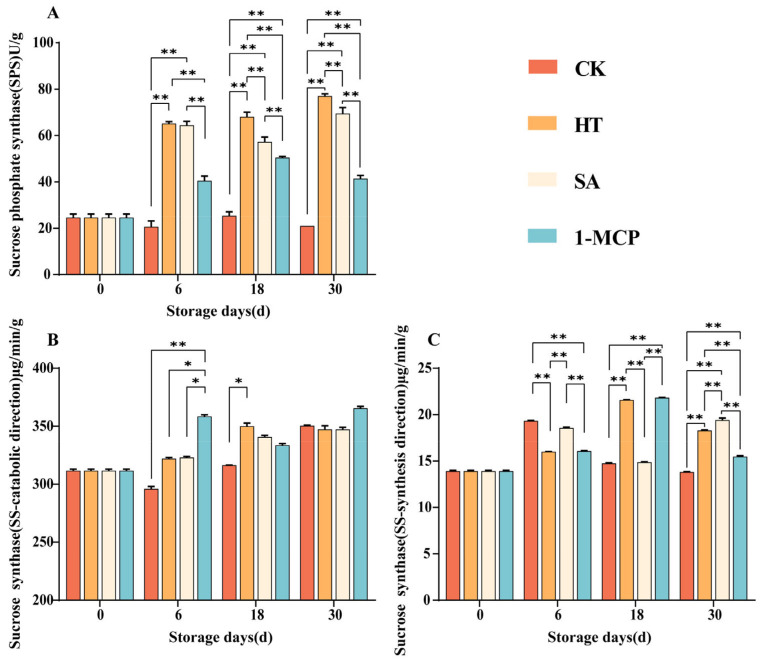
Changes in SPS (**A**), SS synthesis direction (**B**) and SS catabolism direction (**C**) enzyme activities in fruit pulp during storage. “**” means the result of significance analysis is highly significant (*p* < 0.01), “*” means the result of significance analysis is significant (0.01 < *p* < 0.05).

During the postharvest storage period, except for the SPS activity in the control (CK) group, which decreased from 25.73 U/g to 22.01 U/g, the activities of sucrose phosphate synthase (SPS) and sucrose synthase (SS) in the synthesis direction showed a general upward trend with increasing storage duration (Figure 7A,B). This indicates that the activities of enzymes related to sucrose synthesis increased after a period of storage, and that the three treatments enhanced these enzyme activities to some extent. The SS activity in the catabolic direction activity was relatively low, but in the later stages, the activity in the treatment groups was significantly higher than that in CK (*p* < 0.01), indicating that the three treatments increased the activity of SS-catabolism direction enzymes (Figure 7C). On the sixth storage day, when the total sugar content reached its peak, the activities of all three enzymes were elevated, suggesting that enzymes involved in sucrose synthesis might play a major role during this period. On the 30th storage day, the activities of the enzymes related to sucrose synthesis had increased compared to those on day 18, while the activities of enzymes related to sucrose decomposition, except for SA, had decreased compared to those on the 18th storage day. The SPS activity in the treatment groups was significantly higher than that in the CK group at the same time point (*p* < 0.01), which indicated that all three treatments better promoted SS activity in the catabolic direction. The SPS activity of the HT group was significantly higher than that of the SA and 1-MCP (*p* < 0.05) groups, except on the sixth storage day, indicating that heat treatment was the most effective among the three treatments. Overall, the different treatments increased the activities of enzymes related to sucrose metabolism, with a more pronounced effect on the synthesis aspect. This suggested that the treatments could maintain fruit flavor by enhancing the activities of enzymes involved in sucrose synthesis.

### 3.7. Expression of Genes Related to Rate-Limiting Enzymes of Sucrose Metabolism

**Figure 8:**
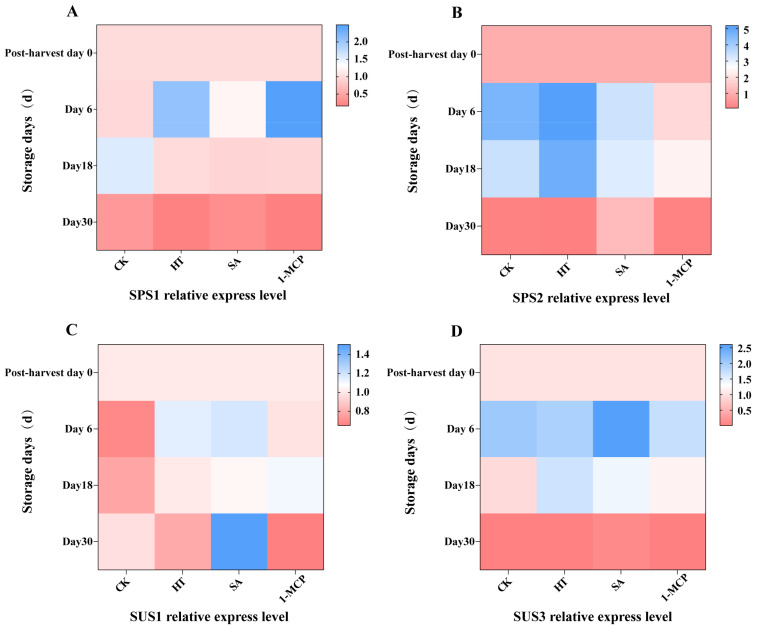
Expression of genes involved in sucrose synthesis and catabolism in Newhall navel orange. (**A**) Expression levels of *SPS1* in navel orange pulp. (**B**) Expression level of *SPS2* in navel orange pulp. (**C**) Expression level of *SUS1* in navel orange pulp. (**D**) Expression level of *SUS3* in navel orange pulp.

The expression peaks of *SPS2* and *SUS3* were relatively high, while those of *SPS1* and *SUS1* were relatively low. This suggested that *SPS2* and *SUS3* might play more important role during the storage process (Figure 8). The expression levels of *SPS1* and *SPS2* exhibited an increasing and then decreasing trend. In the early stage of storage, the expression of *SPS1* and *SPS2* was higher in the treatment groups compared to the control (CK), while in the late stage, their expression levels decreased. It was speculated that exogenous substances might be involved in gene induction through alternative pathways. In the different treatments, the expression of *SUS1* was higher than that in the CK group, while in the late stage, the expression in was lower in all treatments except SA. The expression of *SUS3* also showed an increasing and then decreasing trend. Except for SA, other treatments partially suppressed the expression of this gene in the early stage of storage, which is consistent with the trend of enzyme activity in the SS catabolic direction. However, in the late stage, *SUS3* expression was almost negligible, suggesting that *SUS3* primarily regulates SS catabolic activities. Notably, the SA treatment significantly inhibited the reduction in *SUS1* and *SUS3* expression.

## 4. Discussion

The antioxidant properties of fruits have become a focal point for assessing the quality of fruits and their potential health benefits. In this study, we investigated flavonoids and total phenols as the main antioxidants in ‘Newhall’ navel oranges. Complex changes in the content of these compounds occurred during the different postharvest treatments. Flavonoid content exhibited a trend of initially decreasing and then increasing, with heat treatment (HT) and salicylic acid treatment (SA) showing an advantage in inhibiting the decline in the later stages. This may be due to the activation of certain defense mechanisms in the fruit by these treatments. Heat stress can trigger the synthesis of stress-responsive proteins such as the heat shock proteins (HSPs) involved in flavonoid biosynthesis pathways, as reported in studies on heat-treated fruits [29]. In addition, to counteract the negative effects of heat stress on cells, plants may also stimulate antioxidant enzymes, such as superoxide dismutase (SOD), peroxidase (POD) and catalase (CAT), as well as non-enzymatic antioxidants, such as ascorbic acid and carotenoids [30,31]. However, SA can regulate many aspects of plants at the gene level, thereby increasing their tolerance to abiotic stresses. SA has been reported to induce several genes responsible for encoding chaperones, heat shock proteins (HSPs), antioxidants and secondary metabolites [32].

The three treatments significantly inhibited the decrease in total phenolic content in the different storage periods. Compared with CK, the effect of HT was excellent on day 6 and 30, while SA and 1-MCP performed better on day 18. Which may be related to their different modes of action. Heat treatment likely increased the activity of enzymes involved in phenolic compound synthesis through heat-activated metabolic pathways. In contrast, 1-MCP may enhance antioxidant capacity by interfering with the ethylene signaling pathway [33].

Citrus fruits are high in antioxidants [34], and by quantifying their free radical scavenging ability, we can predict the beneficial effects of citrus on human health. The scavenging capacity for DPPH, ABTS and FRAP radicals decreased over time, with HT showing a slower decrease in scavenging capacity during the late storage period. This might be attributed to the induction of heat shock proteins during heat treatment, which protect cellular components from oxidative damage and maintain the integrity of the antioxidant enzyme system [35].

The weight lost from the fruits increased with storage time. Heat and salicylic acid treatments were more effective in delaying weight loss and maintaining fruit quality. This might be attributed to the following mechanism: heat treatment altered the cell membrane fluidity and integrity, thereby reducing water loss. The salicylic acid treatment enhanced the water potential and increased the plant water content by increasing osmotic pressure [36].

Total soluble solids (TSSs) and titratable acidity (TA) are important parameters affecting fruit quality. Maintaining an appropriate sugar–acid ratio while suppressing the decline in TA content is crucial [37]. Heat treatment significantly increased the total soluble solids content compared to the CK, consistent with the findings of Zhou et al. [38]. Although no significant differences were observed among the treatments in terms of titratable acid content, the SA treatment was sufficient to better stabilize the titratable acid content, which is consistent with findings on the effects of SA on the postharvest storage of table grapes, presumably because SA slows the rate of TA decline by inhibiting respiration rates and ethylene biosynthesis.

Sugar metabolism is a key determinant of flavor and quality during fruit storage. In the ‘Newhall’ navel oranges, the total sugar content initially increased and then decreased, reaching a peak on the sixth storage day. The changes in the soluble sugar content were different under different treatments. HT and SA were more effective in inhibiting the decline of total sugar content during the later storage period. The different treatments exhibited distinct effects on fructose and glucose. Notably, HT had a significant impact on sucrose content (40.51%), which was significantly higher compared to the other three treatments. This finding is similar to previous findings on the regulation of sugar concentration by heat stress in tobacco [39], the significant changes in sugar concentration observed after HT confirm the very complex metabolic response of plants to these abiotic stresses.

Overall, the different treatments increased the activities of enzymes related to sucrose metabolism, with effects on the direction of synthesis mainly in the pre-storage period and on the direction of catabolism throughout the storage period. This suggested that the treatments could maintain fruit flavor by enhancing the activities of enzymes involved in sucrose synthesis. The SPS activity of HT was 68.12 U/g on day 18 and 77.03 U/g on day 30, which was significantly higher than the remaining three treatments, indicating that heat treatment was the most effective among the three treatments. The results for sucrase activity and sucrose content were consistent; heat treatment increased sucrose content by enhancing the activity of enzymes related to sucrose synthesis.

*SPS1* and *SPS2* are genes involved to the regulation of sucrose phosphate synthase SPS, which plays a key role in sucrose synthesis, while *SUS1* and *SUS3* are genes related to the regulation of sucrose synthase SS, which is primarily responsible for sucrose breakdown. The expression levels of *SPS1* and *SPS2* exhibited an increasing and then decreasing trend, indicating that these genes mainly function during the early stages of sucrose synthesis. In contrast, the expression of *SUS3* was almost negligible in the late stage, with *SUS1* being the predominant gene at this time. Sucrose content is known to increase during the ripening stage. While the expression of all invertase isozymes decreased significantly during this period, SPS expression remained higher. Therefore, the accumulation of sucrose at maturity can be attributed to increased SPS activity and reduced invertase activity (vesicle, cytoplasmic and cell wall isoforms). An analysis of SPS1 and SPS2 gene expression showed that the expression of SPS1 in 1-MCP on the sixth day of storage was much higher than that of CK during the same period, and the expression of SPS2 in HT was much higher than that of SA and 1-MCP during the same period. Heat stress has been shown to enhance sucrose content, enzyme expression, proline levels and sugar recovery [40]. Similarly, salicylic acid (SA) treatment has been demonstrated to significantly modulate the transcript abundance of genes related to sucrose biosynthesis and degradation in peach fruit [41]. Norwegian apples treated with 1-methylcyclopropene (1-MCP) exhibited lower sucrose hydrolysis and delayed ripening compared to the control check (CK) group [42]. However, in the present study, 1-MCP treatment was not as effective for the storage of ‘Newhall’ navel oranges. It is hypothesized that this may be due to differences in plant material. ‘Newhall’ navel oranges were used in this experiment, and their growing environment may have determined the effectiveness of the different treatments.

## 5. Conclusions

**Figure 9:**
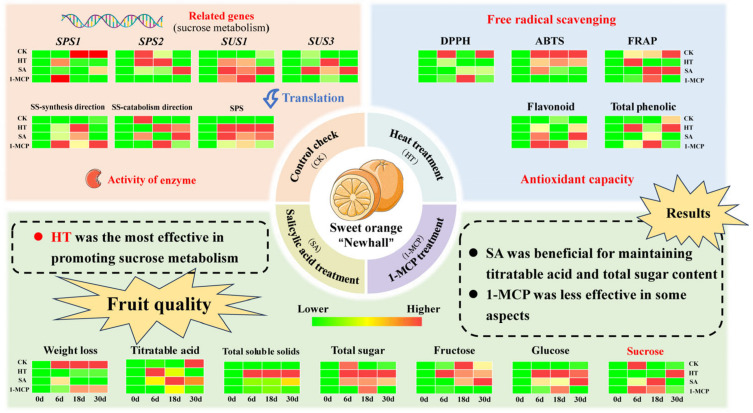
Effects of different postharvest treatments on fruit quality, sucrose metabolism and antioxidant capacity of ‘Newhall’ navel oranges during storage. Different colors represent different values of $\frac{X-X_{\min}}{X_{\max}-X_{\min}}$ (*X* represents the measured value of each index, *X*_min_ represents the minimum value of the corresponding index, and *X*_max_ is the maximum value); higher values are redder and lower are greener.

In this study, we comprehensively investigated the effects of various postharvest treatments, including control check (CK), heat treatment (HT), salicylic acid treatment (SA) and 1-methylcyclopropene treatment (1-MCP) on the quality of ‘Newhall’ navel oranges during storage (Figure 9). HT is often used in postharvest storage management because of its environmental and easy-to-use advantages. HT was the most effective among the three treatments in enhancing the fruit’s antioxidant properties, reducing fruit weight loss, increasing total soluble solids and promoting sucrose metabolism, and it has high application value. SA also showed significant benefits by stabilizing antioxidant capacity, titratable acid and total sugar content, but performed poorly in retarding the decline in total phenolic content and sucrose content. Although the 1-MCP performed the worst in some aspects, it still had a positive effect on antioxidant capacity and sucrose metabolism during storage. These results provide a rationale for selecting postharvest treatments to extend the storage life and maintain the quality of Newhall navel oranges, with broader implications for the citrus industry. However, each of the three treatments has its own strengths in terms of its antioxidant properties, sucrose metabolism and the final quality presented, and no one treatment showed a crushing advantage. Future research could explore combinations of these treatments or develop new approaches to further optimize postharvest fruit quality management to maintain fruit quality and postharvest freshness in citrus fruits.

## Figures and Tables

**Table 1:** Sequence of primers for real-time fluorescence quantification.

Target Gene	Forward Primer (5′-3′)	Reverse Primer (5′-3′)
*SPS1*	TGTAACAGTGGCAGTGAT	GTGTGAGTGGTAATAGAAGTC
*SPS2*	GTTACAACACAAGACACAAT	ATCACCTCAGACCACATT
*SUS1*	GGCCTTTGGCTTGACTGTTG	GGGATCTGCCTTGCACTTCT
*SUS3*	ATTCCGAGCATCAGAGAGCG	TCGTCGATCAGTACATGCGG

**Table 2:** Real-time fluorescence quantitative PCR reaction system.

Ingredient	Volume (µL)
cDNA	1.00
PCR Forward Primer (10 µM)	0.50
PCR Reverse Primer (10 µM)	0.50
2× M5SYBR	6.25
dd H_2_O	4.25
Total	12.50

## Data Availability

Data are contained within the article.
